# Case Report: Anti-platelet factor 4 -mediated immunothrombosis in a patient with ANCA vasculitis – a shared mechanism of NETosis

**DOI:** 10.3389/fimmu.2025.1567999

**Published:** 2025-04-10

**Authors:** Lital Remez-Gabay, Olga Vdovich, Luiza Akria, Etty Kruzel-Davila

**Affiliations:** ^1^ Nephrology Laboratory, Research Institute, Galilee Medical Center, Nahariya, Israel; ^2^ Nephrology Department, Galilee Medical Center, Nahariya, Israel; ^3^ Azrieli Faculty of Medicine, Bar-Ilan University, Zefat, Israel; ^4^ Hematology Unit, Galilee Medical Center, Nahariya, Israel

**Keywords:** anti-PF4 immunothrombosis, NEtosis, ANCA, IVIg, case report

## Abstract

Anti-platelet factor 4 (PF4) immunothrombosis is characterized by thrombocytopenia, thrombosis and enhanced NETosis and has been described in the absence of prior heparin exposure. This case report describes a patient with antineutrophil cytoplasmic antibody (ANCA)-associated vasculitis (AAV) who, while under immunosuppression, developed anti-PF4-mediated immunothrombosis, with NETosis significantly elevated compared to baseline markers observed during AAV. Treatment with intravenous immunoglobulin (IVIG) led to resolution of the syndrome, marked by a reduction in NETosis markers, restoration of platelet counts, and alleviation of the hypercoagulable state. We review the epidemiology, pathogenesis, clinical manifestations, and management strategies of thrombotic anti-PF4 immune disorders, highlighting the roles of AAV and dysregulated NETosis as key triggers. Early recognition of anti-PF4-mediated immunothrombosis without prior heparin exposure is critical, as prompt treatment with IVIG and direct thrombin inhibitors can significantly improve outcomes. This case underscores the interplay between NETosis, ANCA vasculitis, and thrombotic anti-PF4 immune disorders, emphasizing the therapeutic potential of IVIG in mitigating NETosis-related complications.

## Introduction

Heparin-induced thrombocytopenia (HIT) is classified into Type 1, a benign, non-immune reaction resolving spontaneously, and Type 2, a severe immune-mediated disorder caused by anti-PF4-heparin antibodies, leading to thrombocytopenia and thrombosis. Recently, there have been descriptions of anti-PF4-mediated immunothrombosis without prior heparin exposure, expanding the spectrum of thrombotic anti-PF4 immune disorders, beyond classic HIT (1–9). Herein, we report on a patient with antineutrophil cytoplasmic antibody (ANCA) associated vasculitis (AAV) suffering from anti-PF4- mediated immunothrombosis, which developed without prior heparin exposure. We emphasize and investigate the pivotal role of NETosis in mediating the pathogenesis of thrombotic anti-PF4 immune disorders. NETosis is an evolutionarily conserved process aimed to entrap microorganisms (10). NETosis can be triggered by both pathogens and endogenous factors, including antibodies, pro-inflammatory cytokines, and sterile inflammatory stimuli such as high glucose, cholesterol, complement C5a, and hypoxia. Once activated, a cascade of enzymatic events unfolds, beginning with the Raf-MEK-ERK pathway activation and NADPH oxidase-dependent ROS production, followed by an increase in cytosolic calcium, which activates peptidylarginine deiminase 4 (PAD4), leading to histone H3 citrullination (citH3) and chromatin decondensation (11). This process results in the extrusion of a mixture of DNA and bactericidal proteins, including myeloperoxidase (MPO), neutrophil elastase (NE), and citH3, all of which serve as markers of NETosis (11, 12). However, NETosis can also occur independently of NADPH oxidase activity (13), highlighting the existence of both ROS-dependent and ROS-independent pathways (12, 14). While NETosis functions as a vital host defense mechanism, its dysregulation can result in distinct pathological outcomes such as necroinflammation in ANCA-associated vasculitis (AAV) and thrombosis in anti-PF4 immune disorders (15–18). This dual-edged nature of NETosis underscores its critical role in both immunity and pathology.

Crucially, this case study highlights the activation of NETosis and its pivotal role in fueling inflammatory and prothrombotic processes in a patient with AAV and anti-PF4-mediated immunothrombosis. We delve into the epidemiology, clinical features, and proposed mechanisms underlying thrombotic anti-PF4 immune disorders, with a particular focus on NETosis as a key mediator of these complications and its role in AAV pathology. Furthermore, we explore therapeutic implications, emphasizing the role of immunomodulatory treatments such as intravenous immunoglobulins (IVIG) in countering dysregulated NETosis, thrombocytopenia, and thrombosis. We present insights into the management of this complex and rare condition, underscoring the potential of targeted interventions to enhance patient outcomes.

## Case description

**Figure 1:**
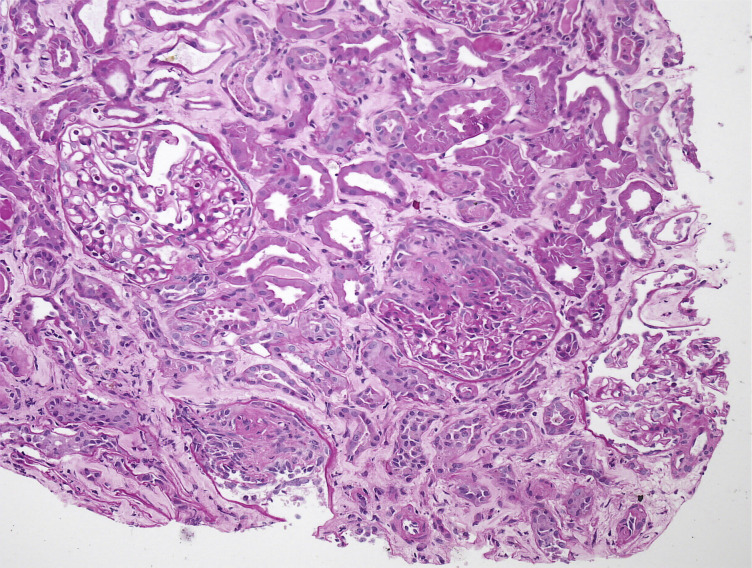
Kidney biopsy demonstrating focal crescentic glomerulonephritis (GN) associated with ANCA.

An 86-year-old woman with a history of hypertension presented with a progressive decline in renal function, with serum creatinine levels rising from 1.4 mg/dL to 7.01 mg/dL over the past four months. On physical examination, she appeared pale, with a respiratory rate of 24 breaths per minute, blood pressure of 154/82 mmHg, and a regular pulse of 88 beats per minute. Cardiac examination revealed normal heart sounds without murmurs or pericardial friction rub, along with bilateral lung crepitations. Her abdomen was soft, non-tender, without organomegaly, and with normal bowel sounds. No peripheral edema was observed. Laboratory evaluation revealed a positive P-ANCA (anti-myeloperoxidase) with a titer of 45.2 AU/mL. Additional laboratory findings are detailed in **Table 1**. A chest CT scan revealed several pulmonary nodules located in the lower anterior region of the right upper lobe and the posterior region of the right lower lobe. A kidney biopsy was performed to confirm rapidly progressive glomerulonephritis (RPGN), revealing crescentic glomerulonephritis due to p-ANCA associated vasculitis. (**Figure 1**). The biopsy sample included 17 glomeruli, of which 3 exhibited fibrocellular crescents, 1 displayed a cellular crescent, and 3 showed global sclerosis. The remaining 10 glomeruli had open capillaries without evidence of hypercellularity. Tubular atrophy affected 10–20% of the sample, with approximately 50% interstitial fibrosis. Notably, no deposition of immunoglobulins or C3 was detected. A diagnosis of crescentic glomerulonephritis secondary to p-ANCA associated vasculitis, specifically microscopic polyangiitis (MPA), was confirmed. The patient received three 1000 mg pulses of methylprednisolone, seven alternate-day plasma exchange sessions, and a single 500 mg dose of cyclophosphamide, resulting in a gradual improvement in renal function, with serum creatinine levels declining to 4.2 mg/dL within one week. Two weeks after starting treatment, the patient developed severe thrombocytopenia, with her platelet count dropping to 5,000/μL (**Table 1**). Stable leukocyte levels indicated that cyclophosphamide was unlikely the cause of this profound thrombocytopenia. A peripheral blood smear showed no evidence of platelet clumping or schistocytes, and follow-up testing revealed that p-ANCA had become negative. (**Table 1**) Six units of platelets were transfused. Twelve hours post-transfusion, the patient developed swelling, cyanosis, and petechiae in her left leg. Doppler ultrasound confirmed deep vein thrombosis (DVT).

**Table 1:** Timeline of laboratory results before, during and after hospitalization.

	Baseline 1.02.2024	Presentation 12.5.2024	Anti -PF4-mediated immunothrombosis 28.5.2024	Discharge 6.6.2024	20.6.2024	22.8.2024	1.10.2024	18.11.2024
**Hemoglobin g/dL**	12	8.5	8.9	8.9	9.4	11.3	12.2	13.1
**Leukocyte count/μL**	7,400	7,730	11.380	4,090	6,700	5,800	4,200	8,400
**Platelet count/μL**	319,000	311,000	5,000	91,000	181,000	251,000	284,000	283,000
**Creatinine, mg/dL**	0.75	7.01	2.6	1.66	1.73	1.27	1.3	1.28
**Albumin, g/dL**	4.3	3.14	3.38	2.87	3.4	4	4	
**CRP, mg/L**		104.5	2.4	6.1				
**LDH. U/L**		201	210	258				
**p-ANCA, AU/mL**		45.2	negative		negative	negative		
**c-ANCA, AU/mL**		negative						
**Urine sediment**		RBC casts	RBC casts	w/o casts	w/o casts	w/o casts		
**Urine protein-to-creatinine ratio, mg/g**		1144				355		469
**Urine albumin-to-creatinine ratio. mg/g**		589				125		148

Hemoglobin (Hb): 12.1–15.1 g/dL, Leukocyte Count (WBC): 4,000–11,000 cells/μL,Platelet Count (PLT): 150,000–450,000 platelets/μL, Creatinine (Cr): 0.6–1.1 mg/dL, Albumin (Alb): 3.5–5.0 g/dL, C-Reactive Protein (CRP): <6 mg/L, Lactate Dehydrogenase (LDH): 120–250 U/L, Perinuclear Anti-Neutrophil Cytoplasmic Antibody (p-ANCA): Negative (<20 AU/mL), Anti-Neutrophil Cytoplasmic Antibody (c-ANCA): Negative (<20 AU/mL), Urine Protein-to-Creatinine Ratio (UPCR): Normal (<150 mg/g), Urine Albumin-to-Creatinine Ratio (UACR): Normal (<30 mg/g).

The combination of severe thrombocytopenia and thrombosis raised suspicion for anti-PF4-mediated immunothrombosis without proximate heparin exposure, given the absence of prior heparin treatment. A rapid immunoassay (STic Expert HIT) detected positive anti-PF4 antibodies in the patient’s plasma, indicating the presence of PF4-specific antibodies, typically associated with HIT. However, the patient had no prior heparin exposure. Platelet Aggregation Test (PAT) using Light Transmission Aggregometry (LTA) with platelet-rich plasma (PRP) and heparin at varying concentrations was performed. According to the reference values of the performing laboratory, a positive aggregation response is defined as greater than 40%. The results demonstrated 23.6% platelet aggregation with buffer alone, 10.1% at 0.1 U/mL heparin, 80% at 1 U/mL heparin, and 0.1% at 100 U/mL heparin. Unlike spontaneous HIT, platelet aggregation was not observed in the absence of heparin. However, similar to HIT, low-dose heparin induced platelet aggregation, while high-dose heparin inhibited aggregation, indicating a heparin-dependent activation pattern. Given that the patient had no prior heparin exposure, these findings suggest that anti-PF4 antibodies, unrelated to heparin, mediated the immunothrombosis (2, 3, 19, 20). Treatment with argatroban, a direct thrombin inhibitor, was initiated with careful monitoring to effectively manage the heightened risk of bleeding associated with severe thrombocytopenia.

### Therapeutic intervention

To manage the immune-mediated thrombocytopenia and thrombosis, IVIG was administered at a dose of 0.5 g/kg daily for 3 consecutive days, while platelet transfusions were avoided to mitigate the risk of exacerbating the prothrombotic state. Notably, a gradual increase in platelet count was evident within 24 hours of initiating IVIG therapy. Once the platelet count reached 70,000/μL, argatroban was transitioned to apixaban for long-term anticoagulation.

### Follow-up and outcomes

The patient’s general condition, kidney function, and platelet counts demonstrated significant improvement over the subsequent 7 days, as detailed in **Table 1**. She is currently receiving maintenance therapy with avacopan (CCX168), a selective complement 5a receptor (C5aR) inhibitor, in combination with rituximab (anti-CD20). Importantly, no further episodes of anti-PF4-mediated immunothrombosis have been observed. Apixaban was discontinued after 6 months. This personalized treatment approach highlights the vital role of early recognition and timely intervention in managing anti-PF4-mediated immunothrombosisin patients without prior heparin exposure, particularly those with AAV.

### Diagnostic assessment

Given the potential link between excessive NET formation and the development of anti-PF4-mediated immunothrombosis (16, 17), we quantified NETosis markers at five critical time points: at the diagnosis of ANCA-associated vasculitis, during the acute phase of anti-PF4-mediated immunothrombosis, and at three intervals following recovery.

### NETosis markers levels in serum

**Figure 2:**
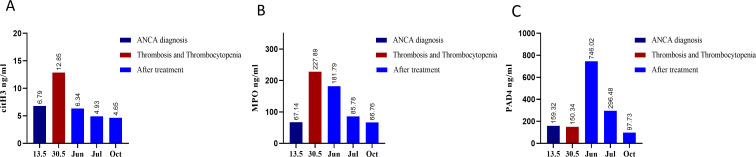
NETosis markers across key stages of disease progression. NET markers were measured by ELISA at five critical points: at the diagnosis of antineutrophil cytoplasmic antibody (ANCA) vasculitis, during the acute phase of anti-PF4-mediated immunothrombosis, and at three intervals following Intravenous Immunoglobulins (IVIG) treatment **(A)** Serum levels of citrullinated histone H3 (citH3) **(B)** Myeloperoxidase serum levels (MPO) **(C)** Peptidyl arginine deiminase 4 (PAD4) serum levels. The data demonstrates a correlation between the reduction in NETosis markers and clinical recovery.

The trends and comparative analysis of these markers are depicted in **Figure 2**, providing insights into the dynamic role of NETosis throughout the disease course and recovery.

**Figure 3:**
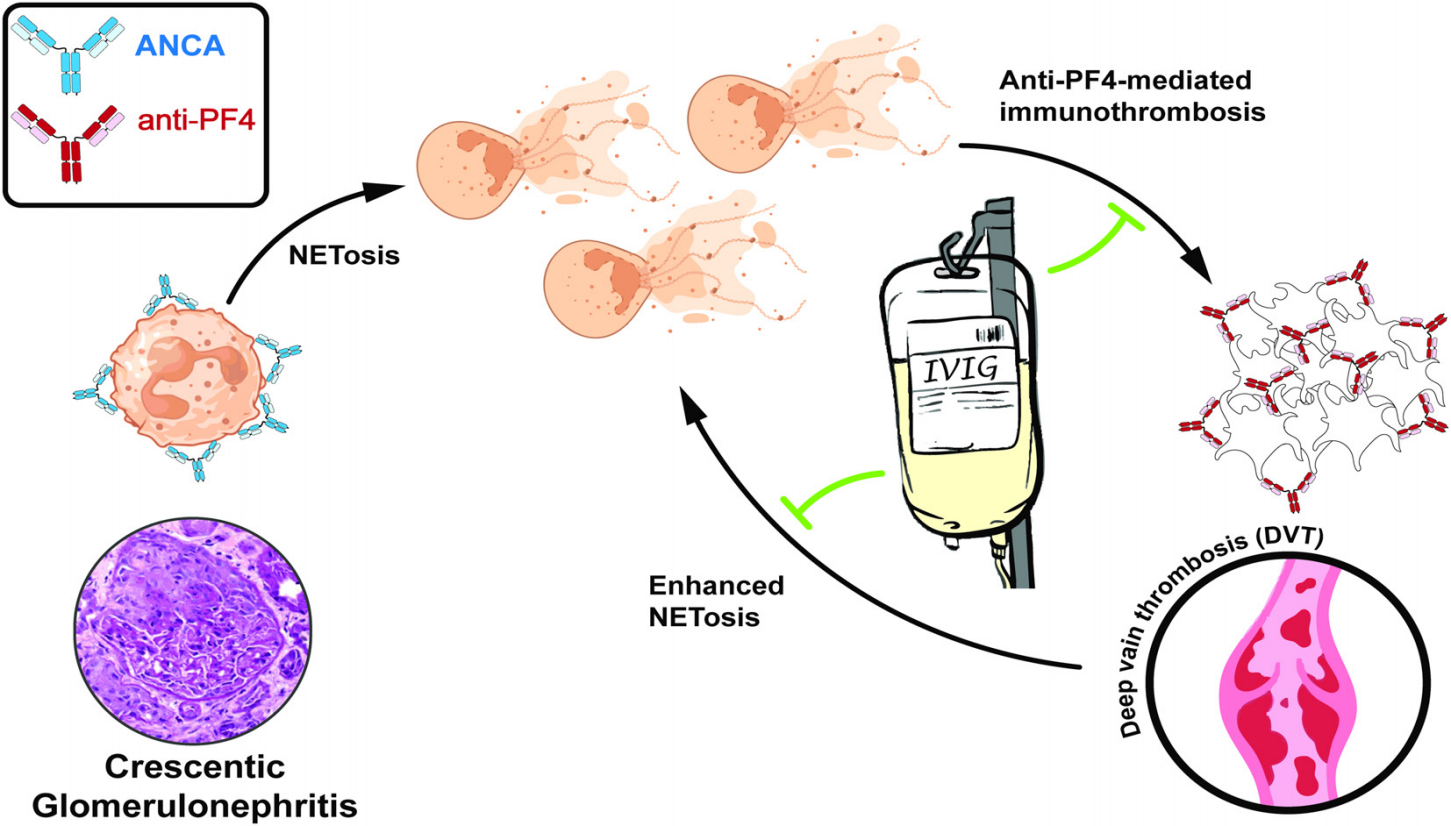
Depiction of NETosis activation in antineutrophil cytoplasmic antibody (ANCA) associated vasculitis (AAV) and anti-PF4-mediated immunothrombosis with IVIG inhibiting dysregulated NETosis and anti-PF4-mediated immunothrombosis complications. NETosis markers were significantly elevated at the time of presentation of rapidly progressive glomerulonephritis (RPGN), with further amplification observed during the complication of anti-PF4-mediated immunothrombosis. Following Intravenous Immunoglobulins (IVIG) treatment, resolution of the anti-PF4-mediated immunothrombosis was achieved, along with improved kidney function and a substantial reduction in NETosis activation.

At the diagnosis of ANCA-associated vasculitis, baseline levels of citH3, MPO, and PAD4 were markedly elevated, reflecting heightened NETosis activity characteristic of active vasculitis. Despite initiating immunosuppressive therapy, including methylprednisolone pulses, plasma exchange, and cyclophosphamide, the patient developed an anti-PF4-mediated immunothrombosis two weeks later. During this complication, citH3 and MPO levels significantly increased, rising 1.9-fold and 3.3-fold, respectively. PAD4 levels exhibited an even more pronounced rise, peaking at a 4.9-fold elevation six days after the acute thrombotic event. These findings underscore the exacerbated NET formation despite ongoing immunosuppressive treatment, highlighting the need for targeted strategies to address dysregulated NETosis in this context. Following the administration of IVIG, a marked reduction in NETosis markers was observed, with levels decreasing to below those measured at the initial ANCA vasculitis diagnosis. This reduction in NETosis markers corresponded with clinical improvement, an increase in platelet count, and resolution of thrombosis, underscoring the efficacy of IVIG in modulating NETosis activity even in the setting of prior immunosuppressive therapy. The dynamic changes in NETosis markers levels across these disease stages are depicted in **Figure 2**, emphasizing the potential of NETosis as a biomarker for disease progression and therapeutic response in ANCA vasculitis and anti-PF4-mediated immunothrombosis. The vicious cycle of dysregulated NETosis in AAV and anti-PF4-mediated immunothrombosis is depicted in **Figure 3**, illustrating its feed-forward pathogenic mechanism. IVIG disrupts this cycle, leading to the attenuation of NETosis, immunothrombosis, and thrombocytopenia, ultimately breaking the cycle of immune-driven thrombosis.

To our knowledge, this is the first description of anti-PF4-mediated immunothrombosis occurring in the absence of prior heparin exposure in association with ANCA-associated vasculitis. This case underscores the importance of clinical vigilance and careful monitoring for anti-PF4-mediated immunothrombosis in patients with ANCA vasculitis, as well as the therapeutic potential of IVIG in managing this condition.

## Discussion

Type 2 HIT is an antibody-mediated, prothrombotic drug reaction first described in 1973, characterized by thrombocytopenia occurring about one week after heparin initiation, frequent thrombotic events, and the presence of heparin-dependent, platelet-activating anti-PF4 antibodies (1, 2, 21, 22). More recently, there have been descriptions of anti-PF4-mediated immunothrombosis without prior heparin exposure, expanding the spectrum of thrombotic anti-PF4 immune disorders, beyond classic HIT (6). Three distinct etiologies of anti-PF4 immune disorders unrelated to heparin exposure have been characterized. 1. Autoimmune HIT (aHIT): A severe subtype of HIT characterized by the presence of both heparin-dependent and heparin-independent platelet-activating antibodies. Unlike patients with classic HIT, patients with aHIT have unusually severe thrombocytopenia, an increased frequency of disseminated intravascular coagulation, and atypical thrombotic events (3, 6). 2. Spontaneous HIT: This syndrome is triggered by non-heparin factors such as knee replacement surgery, infections, or monoclonal paraprotein associations. In these cases, excessive release of PF4 or its binding to other negatively charged molecules can initiate an immune response. This subtype is primarily associated with heparin-independent platelet-activating antibodies (4, 6–8, 23). 3. Vaccine-Induced Immune Thrombotic Thrombocytopenia (VITT): Recently recognized as a rare complication of adenoviral vector-based COVID-19 vaccines, VITT is marked by the presence of high-titer anti-PF4 antibodies. These antibodies induce PF4 clustering and platelet activation in a heparin-independent manner, leading to thrombocytopenia and thrombotic complications. Although antibodies against PF4–heparin and PF4 bind to distinct epitopes on PF4, both types of antibodies cross-link platelet Fc receptors (FcγRIIA), inducing platelet activation and hypercoagulability (5, 6, 9, 24–32).

PF4, also known as CXCL4, is a 32 kDa positively charged tetrameric chemokine protein predominantly stored in and released from platelets, although it can also be synthesized by monocytes. It is regarded as an evolutionarily ancient chemokine with proposed roles in hemostasis and antimicrobial defense, notably through its ability to bind to negatively charged bacteria (26). Polyanionic heparin forms complexes with cationic PF4, inducing conformational changes in PF4 that trigger an immune response. This results in the formation of immunoglobulin G (IgG) complexes that activate platelets and monocytes via FcγIIA receptors. The immune complex-mediated activation triggers a thrombo-inflammatory cascade that transforms endothelial cell surfaces from an anticoagulant to a procoagulant state. This shift occurs through the inhibition of protein C activation and the binding of PF4 to von Willebrand factor, resulting in thrombocytopenia and substantial thrombin generation (1, 2, 6, 26). Risk factors for developing HIT include the use of unfractionated heparin (compared to low-molecular-weight heparin), major surgery, pregnancy, genetic polymorphisms in the FcγRIIa receptor, and blood group O (26). In autoimmune or spontaneous HIT, immunologic “danger signals” during inflammation or trauma can increase the likelihood and intensity of forming an anti-PF4 immune response (26). The binding forces of the anti-PF4/polyanion autoantibodies exceed even the binding force of long heparin molecules to fuse together two PF4 tetramers mimicking heparin induced platelets aggregation (3, 6, 26). Moreover, a subset of antibodies from autoimmune HIT patients have high affinity to PF4 alone in the absence of polyanions. The resulting immune complexes induce massive platelet activation in the absence of heparin (5, 25–29, 33).

In addition to platelet activation and endothelial injury mediated by PF4 antibodies, another mechanism contributing to thrombosis in thrombotic anti-PF4 immune disorders involves the activation of NETs (16). Excessive or dysregulated NET formation can lead to tissue damage, necroinflammation, and the development of a hypercoagulable state (10, 12, 34). NETs not only promote thrombin generation but also harbor prothrombotic components, including tissue factor, protein disulfide isomerase, factor XII, von Willebrand factor (VWF), and fibrinogen. Furthermore, platelet-neutrophil interactions mediated by P-selectin stimulate additional NET formation, creating a feedback loop that amplifies thrombosis (12, 16).

Anti-PF4 antibodies including HIT immune complexes and VITT antibodies directly induce NETosis by engaging FcγRIIA receptors on neutrophils and facilitating neutrophil-platelet interactions. These immune complexes drive the formation of thrombi containing neutrophils, extracellular DNA, citH3 and platelets, which are consistent markers of NETosis. This has been demonstrated in both microfluidic systems and *in vivo* studies, where neutrophil depletion completely prevented thrombus formation. In HIT models, such as FcγRIIA+/hPF4+ mice treated with HIT IgG antibodies, inhibition of PAD4 abolished thrombus formation. Furthermore, NETs markers and neutrophils undergoing NETosis have been identified in the plasma of patients suffering from HIT or VITT, further supporting the role of NETosis in driving thrombosis in HIT and HIT-like syndromes (16–18, 35–37).

The pathogenesis of AAV involves ANCA-mediated neutrophil activation, resulting in the release of inflammatory cytokines, reactive oxygen species (ROS), and the formation of NETs (15). While there have been reports of co-occurrence of AAV and HIT, it is important to emphasize that this represents the coexistence of two distinct disorders rather than a shared or common pathogenic mechanism (38–40). Given the shared pathomechanism of dysregulated NETosis in both AAV and anti-PF4-mediated immunothrombosis, we hypothesize that dysregulated NETosis, as well as impaired NET clearance, may underlie the co-existence of these two conditions in our patient. It is well-established that polyanions, such as DNA and RNA, released during dysregulated NETosis, can form antigenic complexes with PF4 (41, 42). This raises the possibility that pharmacologically active aptamers released during NETosis in AAV patients might act as potential triggers for anti-PF4-mediated immunothrombosis. Interestingly, in our patient, the anti-PF4-mediated immunothrombosis developed despite ongoing immunosuppressive therapy and responded to IVIG treatment. This was evidenced by clinical improvement, including increased platelet counts, inhibition of thrombosis propagation, and a marked decrease in NETosis markers. These findings suggest that IVIG may exert a specific inhibitory effect not only on platelet activation but also on NETosis. This further implicates dysregulated NETosis as a central mechanism driving the development of anti-PF4-mediated immunothrombosis in this context.

The diagnosis of spontaneous anti-PF4-mediated immunothrombosis involves confirming the presence of anti-PF4 antibodies, typically using immunoassays. Platelet function is then evaluated through functional assays such as Heparin-Induced Platelet Aggregation (HIPA) test or other PATs and the serotonin release assay (SRA). In HIT, high platelet aggregation at low heparin concentrations, followed by inhibition at supraphysiologic heparin doses, confirms the diagnosis. Similar findings are observed in spontaneous HIT; however, a key distinguishing feature is that spontaneous platelet aggregation occurs even in the absence of heparin, reflecting a heparin-independent platelet activation mechanism (3, 19, 43). The inhibitory effect observed at higher heparin concentrations is attributed to the capacity of excess heparin to bind individual PF4 molecules, thereby disrupting the close approximation of PF4 tetramers necessary for the formation of platelet-activating PF4/IgG immune complexes (3, 19, 43). In addition, use of PF4, rather than heparin, also optimized reactivity for detecting VITT antibodies, in the HIPA-modified assay known as the “PIPA” (43).

Standard HIT treatments, such as discontinuing heparin, are not applicable in anti-PF4-mediated immunothrombosis without prior heparin exposure. Management relies on anticoagulants, including direct thrombin inhibitors (argatroban, bivalirudin) and factor Xa inhibitors (danaparoid, fondaparinux, rivaroxaban) (6). A key challenge with direct thrombin inhibitors is their monitoring via activated partial thromboplastin time (PTT), which can be misleading in cases of concurrent disseminated intravascular coagulation (DIC), potentially leading to underdosing. Careful interpretation of PTT and alternative monitoring strategies are crucial (6). Switching to oral anticoagulants like apixaban may be appropriate once platelet levels stabilize. In severe cases, antibody-induced -platelets activation can be rapidly mitigated through high-dose IVIG therapy. *In-vitro* studies have demonstrated that IVIG inhibit platelet-activating properties of aHIT sera, independent of heparin (6, 44). The mechanism of action of high-dose IVIG in treating anti-PF4-mediated immunothrombosis may involve neutralizing anti-PF4 antibodies, thereby blocking their ability to activate platelets by inhibiting FcγIIA receptor engagement. Additionally, it has been proposed that high-dose IVIG may competitively inhibit FcγRIIA-mediated platelet activation by increasing plasma IgG levels, which outcompete pathogenic antibodies for receptor binding (44). IVIG can have dual effects on NETosis. On one hand, Fc-gamma receptor (FcγR) crosslinking can act as a stimulus for NET release (45). On the other hand IVIG has been demonstrated to induce a dose-dependent abrogation of NET production *in-vitro* and significantly reduce NETosis markers in COVID-19 patients treated with IVIG (46). In our case, the attenuation of NETosis following IVIG treatment supports the latter mechanism. These findings suggest that IVIG treatment provides therapeutic benefits in conditions characterized by excessive NET formation, including severe inflammatory and thrombotic states like anti-PF4-mediated immunothrombosis (6, 44, 46).

## Limitation

We did not perform the HIPA assay, however, to address this limitation, we conducted a Platelet Aggregation Test (PAT) using Light Transmission Aggregometry (LTA) with platelet-rich plasma (PRP) and heparin at varying concentrations, as a modified approach resembling the HIPA test. The absence of spontaneous platelets aggregation in the buffer control, does not fully align with typical descriptions of spontaneous HIT in the literature, although documented exceptions do exist (19, 20, 43). While the result in buffer was negative, the HIPA-modified assay known as the PF4-Induced Platelet Activation (PIPA) test, which utilizes PF4 instead of heparin and enhances the detection of certain platelet-activating anti-PF4 antibodies, was unfortunately not available to us. Nevertheless, unlike most VITT-associated antibodies, the patient’s serum tested positive for anti-PF4 antibodies using the rapid immunoassay (STic Expert HIT) and also demonstrated a positive PAT result using LTA with PRP and heparin at varying concentrations, as described in the Methods section (8, 19, 20, 43).

Another limitation of our study is the lack of well-defined reference ranges for citH3, MPO, and PAD4 in healthy individuals, which prevents the use of absolute cutoff values to define their elevation. Instead, we relied on relative changes correlated with disease activity, supporting their potential role in disease pathophysiology.

Additionally, we did not conduct functional studies to establish a direct mechanistic link between ANCA-PF4 interactions and NETosis induction, nor did we provide experimental validation of IVIG’s effects on NET formation and clearance. Future research is needed to explore these mechanisms in-depth and confirm the proposed associations.

## Conclusion

Anti-PF4-mediated immunothrombosis without prior heparin exposure is a rare clinical condition that requires high clinical suspicion, especially in patients with inflammatory or autoimmune conditions like AAV. This case underscores the importance of early intervention with IVIG and non-heparin anticoagulation to improve outcomes and reduce complications. Additionally, the possible involvement of NETosis in its pathogenesis presents a potential therapeutic target. Future research should investigate NET inhibitors, such as PAD4 inhibitors, as a possible strategy to manage spontaneous anti-PF4 immune disorders and prevent severe thromboembolic complications.

### Patient perspective

The patient reports a good quality of life, with no symptoms of her disease and no side effects from the immunomodulation therapy.

#### Methods

Rapid immunoassay (STic Expert HIT/Stago) was used to detect anti-PF4 antibody.

A Platelet Aggregation Test (PAT) using Light Transmission Aggregometry (LTA) with platelet-rich plasma (PRP) and heparin at varying concentrations was performed as a modified HIPA test. The test was conducted by mixing donor platelet-rich plasma with buffer and heparin (0, 0.1, 1, and 100 U/mL) and incubating for 15 minutes at 37°C. The patient’s plasma was then added and incubated for an additional 30 minutes at 37°C. Platelet aggregation was assessed using LTA, evaluating heparin-dependent platelet activation.

#### Blood sample collection

Serum samples were collected at five key time points: at AAV diagnosis, during the acute phase of anti-PF4-mediated immunothrombosis and at three intervals post-recovery. Blood was drawn into clot activator tubes, centrifuged at 3000 rpm for 10 minutes at 4°C, and the serum was immediately stored at -80°C for subsequent analysis.

#### Quantification of NET markers in serum by ELISA

All serum samples were diluted 1:5 and citrullinated histone H3 (citH3) was quantified using the Citrullinated Histone H3 ELISA Kit (501620, Cayman). Serum was diluted 1:10 for MPO measurement using Human Myeloperoxidase ELISA Kit (ELH-MPO, RayBiotech). PAD4 was detected in serum (1:5 dilution) using Human PADI4 ELISA Kit (ELH-PADI4, RayBiotech) according to the manufacturer’s instructions. Graphs were generated using Prism software version 2.1.

## Data Availability

The raw data supporting the conclusions of this article will be made available by the authors, without undue reservation.
